# Potential role of phytochemicals in brain plasticity: Focus on polyunsaturated fatty acids

**DOI:** 10.20463/pan.2020.0003

**Published:** 2020-03-31

**Authors:** Jang Soo Yook, Minchul Lee

**Affiliations:** 1 Center for Functional Connectomics, Brain Science Institute, Korea Institute of Science and Technology (KIST), Seoul Republic of Korea; 2 Department of Sports Medicine, CHA University, Pocheon Republic of Korea

**Keywords:** brain, exercise, functional food, gut-microbiome, omega-3, phytochemicals

## Abstract

**[Purpose]:**

Functional foods are thought to strongly influence the structure and function of the brain. Previous studies have reported that brain-boosting diets may enhance neuroprotective functions. Certain foods are particularly rich in nutrients like phytochemicals that are known to support brain plasticity; such foods are commonly referred to as brain foods.

**[Methods]:**

In this review, we briefly explore the scientific evidence supporting the neuroprotective activity of a number of phytochemicals with a focus on phenols and polyunsaturated fatty acids such as flavonoid, olive oil, and omega-3 fatty acid.

**[Results]:**

The aim of this study was to systematically examine the primary issues related to phytochemicals in the brain. These include (a) the brain-gut-microbiome axis; (b) the effects of phytochemicals on gut microbiome and their potential role in brain plasticity; (c) the role of polyunsaturated fatty acids in brain health; and (d) the effects of nutrition and exercise on brain function.

**[Conclusion]:**

This review provides evidence supporting the view that phytochemicals from medicinal plants play a vital role in maintaining brain plasticity by influencing the brain-gut-microbiome axis. The consumption of brain foods may have neuroprotective effects, thus protecting against neurodegenerative disorders and promoting brain health.

## INTRODUCTION

The brain, arguably the most complex structure in the human body, can be thought of as the control tower of the body; it comprises neurons and neuroglia, which serve to selectively route signals that underlie specific cognitive functions^1^. Cognitive functions, including learning and memory, are influenced by a variety of factors that include aging, stress, enrichments in the environment, and physical exercise^2-4^. We refer to this phenomenon as brain plasticity, a process in which nutrition intake plays a critical role.

The brain, which is an energy-intensive organ, consuming about 20% of the body's calories, requires consistent food intake to maintain proper health^5^. Recent evidence also indicates that modern diets may lead to metabolic diseases^6^. These findings therefore suggest that suitable diets, which are linked to brain health and neurodegenerative disorders, are required to maintain proper focus throughout the day. Scientists are subsequently recognizing the strong link between food and brain health.

In this review, we briefly assess the efficacy of phytochemicals, focusing on their neuroprotective actions involving changes at the structural, functional, and molecular levels that might contribute to brain plasticity. Neuroprotective actions include the ability of the central nervous system (CNS) to recover from disorders or injuries and ameliorate the effects of alterations in the structures of synapses and neurons due to internal as well as environment changes. Thus, the identification of phytochemical compounds and their multiple targets is a potentially promising therapeutic strategy for promoting health. In addition, the activation of brain plasticity in response to various stressors, by stimulating specific signal transduction pathways and transcription factors, is also discussed.

### The brain-gut-microbiome axis

The brain and intestines communicate with each other in both directions. Food is broken down into its constituent nutrients, absorbed into the bloodstream, and transported to the brain. It provides the energy to the depleted brain, activates cellular responses, and contributes to the maintenance and function of brain tissue^7^. This means that every time a message is sent from the brain to the gut microbiome, messages, in turn, ascend from the gut to the brain; this communication between the brain and the gut is referred to as the brain-gut-microbiome axis.

**Figure 1.**
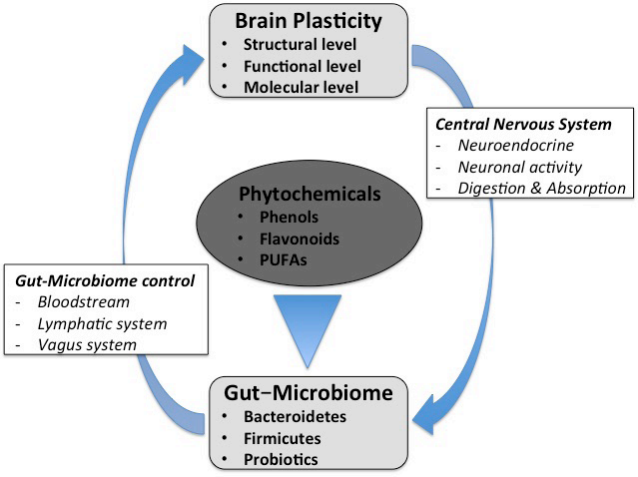
The bidirectional brain-gut-microbiome connections and their relation to phytochemicals. Illustration of how phytochemicals from medicinal plants play a vital role in maintaining brain plasticity by influencing the brain-gut-microbiome axis.

The microbiome plays an important role in the absorption of nutrients from digested food in the gut. It has been shown that the gut microbiome is involved in the digestion of food, production of nutrients, and the delivery of the nutrients to various organs^8^. Messages from the gut microbiome to the brain are dynamic. Bacteria in the microbiome promote the ability of enzymes to break down nutrients in food and convert them into brain hormones or neurotransmitters that the body needs^9^. Therefore, enteric beneficial bacteria like probiotics play a key role in determining health and longevity.

The gut microbiome controls the way our bodies respond to stress^10^. Messages are sent and received by the brain through to the bloodstream and the lymphatic system, they and are also transmitted to the vagus nerve through the intestinal beneficial bacteria's close connection with mitochondria. Fatty acids provide mitochondria with an easy access to fuel^11^. This enhances ketosis, which is known to alleviate cognitive impairment^12^. Mitochondria are responsible for cell signaling, cell death, cell growth, and cellular differentiation, which determines which cell type a given cell changes into, in addition to producing the energy needed for the cell.

There are many different types of bacteria, largely divided into two groups. Bacteroidetes are good bacteria and are the dominant group of bacteria that live in our bodies. Firmicutes are another group of bacteria that are not dangerous but at higher concentrations can overpower and control Bacteroidetes^13^. In the gut, both beneficial and harmful bacteria are affected by ingested foods, which when properly balanced, become symbiotic. The main function of the intestinal microbiome is to create, control, and maintain intestinal barriers. Intestinal microbiome is also involved in vitamin synthesis, metabolism, and glycemic control, and it also affects the expression of genetic information and the production of brain chemicals^7^.

In recent years, the interest in the gut microbiome has led to an interest in the impact of diet on the brain^8^. The benefits of a high fiber diet in the colon have been well documented in epidemiological studies, but its potential impact on the brain has largely been understudied. Taken together, previous results indicate that the brain and intestines might be strongly connected, and that the microbiome is affected by the food in the gut. It is therefore important to ingest foods that are beneficial to gut microorganisms in order to promote brain health.

### The effects of phytochemicals on gut microbiome and their potential role in brain plasticity

Phytochemicals are compounds that are produced by plants. Polyphenols are plant compounds found in many natural foods. There are over 4,000 types of polyphenols, including flavonoids and phenols that are found in fruits, vegetables, grains, beans, and other plants, which are responsible for a variety of colors, tastes, and aromas^14^. Phytochemicals are tasked with providing protection to plants against oxidative stress and inflammation, and are particularly concentrated in plants. These novel compounds have been shown to play a much more important role in human health than previously thought. Some phytochemicals are believed to protect cells from damage that could lead to cancer^14^. The term ‘phytochemicals’ is generally used to describe plant compounds that are being studied for their effects on human health, and are not scientifically defined as essential nutrients.

Similarly, polyphenols include many antioxidants that prevent aging^15^. They also promote autophagy, improve cognitive performance, and eliminate free radicals from the body^16^. Probiotics are closely related to polyphenols. Resveratrol is a powerful polyphenol, and one of the reasons why Mediterranean diets are good for intestinal and brain health is their richness in polyphenols, which have been shown to protect against neuroinflammation^17^.

### The role of polyunsaturated fatty acids in brain health

Polyunsaturated fatty acids are the most abundant fatty acids in cell membranes in the brain^18^. The brain is designed to collect these fats through dedicated entrances located within the vascular brain barrier, so that numerous polyunsaturated fatty acids constantly flow into the brain.[19] Indeed, the brain needs polyunsaturated fatty acids to form the larger and more complex phospholipids and sphingophospholipids mentioned earlier.

Of all the polyunsaturated fatty acids (PUFAs), the two best known to promote brain health are omega-3 fatty acids and omega-6 fats. The same omegas function completely differently, so acquiring both of these polyunsaturated fatty acids through daily diets is recommended. PUFAs and their mediators regulate several processes within the brain, such as neurotransmission, cell survival, and neuroinflammation, thereby regulating mood and cognition. PUFA levels and the signaling pathways that they regulate are altered in various neurological disorders, including Alzheimer's disease and major depression^20^.

Omega-3 fatty acids are particularly important for maintaining proper cardiovascular health through their anti-inflammatory properties^21^. There are three main types of omega-3 fatty acids obtained from food—alpha linoleic acid (ALA), eicosapentaenoic acid (EPA), and docosahexaenoic acid (DHA). Among these, ALA is abundant in marine sources such as spirulina and in vegetable sources, especially in olive oil^22^. A recent study of about 400,000 people over the age of 16 years showed that people who consume high amounts of fish and long-chain omega-3 fatty acids had significantly lower overall mortality, as well as lower cardiovascular and respiratory mortality^19^. Patients with high omega-3 indices showed increased blood flow in areas of the brain associated with learning, memorization, and depression avoidance, and in those with low omega-3 indices, these effects were reversed^23^. In addition, women who consumed high levels of omega-3 were shown to have larger hippocampus and overall brain sizes, and better memory, compared to women who consumed lower amounts of omega-3^24^.

Mediterranean diet refers to the traditional diet of people residing in the Mediterranean region. Extra virgin olive oil, in particular, is a popular Mediterranean food that can dramatically reduce the incidence of, and risks associated with, dementia and other neurological diseases^25^. Olive oil has long been thought of as a healing food for health and longevity. A previous study showed that monkeys fed a Mediterranean diet for two years had a much wider variety of microbial genomes and higher rates of beneficial bacteria than harmful bacteria compared with monkeys fed a Western diet^26^.

Olive oil is a rich source of oleic acid, an unsaturated fatty acid; however, this fatty acid is not the cause of heart disease, cognitive decline, Alzheimer's disease, and neurological inflammation^27^. In fact, the polyphenol component of olive oil has a positive influence on health. These plant compounds stimulate autophagy so that cells can be recycled. Olive oil has long been known for its high anti-inflammatory effects due to its high polyphenol content^28^. Polyphenols also prevent intestinal microbes from making trimethylamine N-oxides that damage blood vessels^29^.

Indeed, rats fed a diet rich in olive oil had higher levels of autophagy than rats fed a normal diet, and performed better in memory and learning tests than whose diets lacked olive oil. In addition to autophagy, mice that were fed olive oil also had low levels of amyloid plaques in their brains. A diet high in olive oil stimulates nerve cells in the brain stem to secrete the glucagon-like peptide-1 (GLP-1) hormone, which has been reported to lower blood sugar levels and reduce the risk of weight loss or hypoglycemia^30^. GLP-1 also protects against synaptic activity, protecting synaptic axons and dendrites from amyloid toxicity^31^.

### The effects of nutrition and exercise on brain function

A study evaluating brain function in three groups, before and after each group ate diets rich in either olive oil or walnuts, or a low-fat diet, respectively, showed that memory and cognition decreased significantly in the low-fat diet group, while the nut-fed group exhibited significantly improved memory^32^. In another experiment, the diets of more than 400 elderly people were examined and brain images were recorded for three years to determine the effects of eating habits on brain health. The results showed that those who consumed high amounts of olive oil, and ate lower amounts of fried foods or red meat, had about 50% fewer brain contractions^33^. These findings suggest that eating habits strongly influence brain health.

Proper brain function is achieved in part by promoting the production of the brain-derived neurotrophic factor (BDNF), a beneficial protein that maintains the growth and connectivity of dendrites and axons^34^. BDNF promotes neuronal growth, particularly if they are not damaged by inflammation, resulting in improvements in long-term memory and cognition. Research on olive oil and brain health have yielded results that are remarkable enough to suggest the use of olive oil as a remedy to avoid or slow dementia^35^

Furthermore, exercise has been shown to increase BDNF production, prevent neurodegeneration in old age, and protect cells from inflammation^36,37^. Exercise increases BDNF expression, which improves cognitive function, and consequently helps brain health and creates a better environment for new neurons to grow^38^. These results suggest that the effects of exercise is related to the brain-gut axis, linking proper diets with exercise neuroscience^39^.

## CONCLUSION

In this article, we discussed the potential advantages of brain food, which we believe provides useful information about maintaining proper brain health. The medical community has recommended dietary adjustments as part of treatment plans for various diseases such as obesity, diabetes, hypertension, and hyperlipidemia. However, few dietary recommendations have been made to prevent brain aging and dementia. Diets may play an important role in how our brain ages and may be involved in the risk of the development of brain diseases. A variety of actions is needed for addressing the wide range of imbalances that often occur in neurological disorders. Additionally, more research is needed to determine which functional foods may offer benefits in brain plasticity.

